# Chylous Ascites in Bright's Disease

**Published:** 1909-02-06

**Authors:** 


### CHYLOUS ASCITES IN BRIGHT'S DISEASE.

Chylous ascites and chyluria are terms which are apt to conjure up at once the idea of elephantiasis. The latter is doubtless the chief cause of chyluria; but it is not extremely uncommon to meet with milky ascitic fluid in other diseases, notably in acute or subacute tubal nephritis. The following is an example of such chylous ascites in the large white kidney variety of chronic Bright's disease.

The patient was a man aged 41, a cook by trade, who came under observation in hospital for nephritis. He had had smoky urine for six weeks before admission, and he complained of pains in the region of his right kidney. There was a very large amount of albumin in the urine, with many renal tube casts and epithelial cells, and a small quantity of blood. The heart, lungs, alimentary and nervous system seemed natural. There had been no syphilis.

Treated in the ordinary way there was some relief, but the patient was discharged one January with an abundance of albumin in his urine still. Nine months later—in September—he returned with increased albuminuria and general anasarca. Little urine was being passed. Purgatives, diuretics, and milk diet made little difference to the condition. The ascites increased, and as there was also considerable dyspnœa, it became advisable to perform paracentesis abdominis. The fluid which came away looked just like thin milk. It is noteworthy that it had little or no smell, even after it had been left to stand for several days.

The patient lived only two months longer. An autopsy was performed, and typical large white kidneys were found. The following analysis of the ascitic fluid is fairly typical of what is generally found in these cases:—The reaction was neutral, and it remained so even after several days' exposure to the air, during which time there was no spontaneous coagulation, no change of colour, no smell, and no obvious decomposition. The specific gravity was 1008.

Microscopically the chief peculiarity noted in the urine was the abundance of minute granules or particles of fat; there were no large fat globules. There were altogether 4 parts of fat per 1,000 of fluid. No cholesterin was present. After slight acidulation there was a marked coagulum of albumin on boiling, the amount of coagulable proteid being 7.2 parts per 1,000.

There was also a certain amount of non-coagulable proteid present in the form of albumoses or peptones. Urea was present, as demonstrated by the formation of crystals of urea nitrate. With sodium hypobromite there was effervescence, the urea amounting to nearly 1.5 parts per 1,000. The inorganic salts—chlorides, sulphates, and phosphates—together amounted to 8.9 parts per 1,000, of which chlorides were responsible for 6.4.

